# Minimal mitochondrial respiration is required to prevent cell death by inhibition of mTOR signaling in CoQ-deficient cells

**DOI:** 10.1038/s41420-021-00591-0

**Published:** 2021-08-04

**Authors:** Ying Wang, Siegfried Hekimi

**Affiliations:** Department of Biology, McGill University, Montreal, QC Canada

**Keywords:** Cell biology, Drug discovery

## Abstract

Coenzyme Q (CoQ) is a lipid-like mobile electron transporter of the mitochondrial respiratory chain. Patients with partial loss-of-function mutations in the CoQ biosynthesis pathway suffer from partial primary CoQ deficiency (MIM 607426). This leads to mitochondrial dysfunction, which presents like mitochondrial disease syndrome (MDS). In addition, many other conditions, including MDS itself, lead to secondary CoQ deficiency. We sought to identify drugs that can alleviate the consequences of the mitochondrial dysfunction that is associated with CoQ deficiency. Loss of the CoQ-biosynthetic enzyme COQ7 prevents CoQ synthesis but leads to the accumulation of the biosynthetic intermediate demethoxyubiquinone (DMQ). *Coq7-*knockout mouse embryonic fibroblasts (MEFs) die when rapid ATP generation from glycolysis is prevented. We screened for drugs that could rescue cell death under these conditions. All compounds that were identified inhibit mTOR signaling. In the CoQ-deficient cells, the beneficial action mTOR inhibition appears to be mediated by inhibition of protein translation rather than by stimulation of autophagy. We further studied the *Coq7-*knockout cells to better determine under which conditions mTOR inhibition could be beneficial. We established that *Coq7-*knockout cells remain capable of a low level of mitochondrial respiration mediated by DMQ. To obtain more profound mitochondrial dysfunction, we created double-knockout mutant MEFs lacking both *Coq7,* as well as *Pdss2*, which is required for sidechain synthesis. These cells make neither CoQ nor DMQ, and their extremely small residual respiration depends on uptake of CoQ from the culture medium. Although these cells are healthy in the presence of sufficient glucose for glycolysis and do not require uridine or pyruvate supplementation, mTOR inhibitors were unable to prevent their death in the absence of sufficient glycolysis. We conclude that, for reasons that remain to be elucidated, the energy-sparing benefits of the inhibition of mTOR signaling require a minimally functional respiratory chain.

## Introduction

Coenzyme Q (CoQ), also known as ubiquinone, carries electrons in the respiratory chain [1]. CoQ comprises a redox-active benzoquinone head with a polyisoprenoid sidechain of species-specific length (nine or ten subunits in mice and ten in humans). The intracellular CoQ pool relies on endogenous synthesis. Over a dozen nuclear genes are known to participate in eukaryotic CoQ biosynthesis [2–4], whose first step is the synthesis of the isoprenoid sidechain, catalyzed by PDSS1/2. The enzyme COQ7 is responsible for the penultimate step of the CoQ biosynthetic pathway. *Coq7* mutants accumulate demethoxyubiquinone (DMQ), the only stable biosynthetic intermediate [5–9]. Targeted germline deletion of *Coq7* in mice results in embryonic lethality [5, 7], but animals survive for several months after induced adult knockout, despite severe phenotypes and an early death [10]. Whether DMQ has any biological function in vivo is unclear and has been controversial [7, 11–15].

Patients with mutations in genes required for the biosynthesis of CoQ suffer from inborn primary CoQ_10_ deficiency (MIM 607426) [6, 16, 17]. In the last few years, the wider availability of genetic screening and testing has led to the diagnosis of an increasing number of patients with this syndrome [18]. Like many other mitochondrial disorders, primary CoQ_10_ deficiency is associated with clinically heterogeneous symptoms, including infantile multisystem disorder, encephalopathy, mitochondrial myopathy, nephrotic syndrome, and cerebellar ataxia [6, 17, 19–35]. Low CoQ levels are also frequently observed in mitochondrial disease syndrome patients without primary deficiency and in some age-related diseases (e.g., Parkinson disease) [4, 36–40]. Oral CoQ_10_ supplementation is the only treatment option for low CoQ, but the reported efficacy is highly variable and generally unsatisfactory [41].

The main biochemical consequence of CoQ deficiency is impairment of mitochondrial respiration. Although there has been a great interest in developing therapies to treat mitochondrial dysfunction, no such treatment is yet clinically available [42, 43]. One of the proposed approaches for alleviating energy stress is the inhibition of the mammalian/mechanistic target of rapamycin (mTOR). mTOR is a cytosolic Ser/Thr kinase belonging to the phosphatidylinositol kinase‐related family of protein kinases. It plays a central role for adaptation to environmental conditions by regulating the balance between anabolic processes such as the biosynthesis of proteins, lipids, and organelles, and catabolic processes such as autophagy [44]. Rapamycin, the most studied mTOR inhibitor, is reported to be beneficial in models of mitochondrial dysfunction, including the *Ndufs4* knockout (similar to Leigh syndrome), a model for mitochondrial myopathy (the Deletor mouse), a muscle-specific conditional knockout for the complex IV assembly factor *Cox15*, a mouse model with mtDNA depletion syndrome, complex I-deficient *C. elegans gas-1* mutants, and cells exposed to mitochondrial inhibitors [45–50]. For CoQ deficiency, both failure and partial success of rapamycin treatment have been reported in different models [50, 51].

Primary CoQ deficiency is different from other respiratory chain (RC) defects in that the organization of the RC remains intact while only electron flow is impeded. Thus, RC deficits from CoQ deficiency could be more amenable to pharmacological intervention. In the present study, we sought to identify drug candidates that could relieve the deleterious consequences of CoQ deficiency on respiratory function by screening compounds on mouse *Coq7*-knockout (KO) cells. In these cells, the CoQ biosynthetic pathway is intact, except for the two last steps, and as a result, they accumulate DMQ instead of CoQ [7, 11]. Like *COQ* patient cells, *Coq7* KO cells have intact respiratory chain assemblies but low electron transport. Patient cells, however, have residual CoQ_10_ levels, which allows them to maintain sufficient mitochondrial function to survive in medium (galactose instead of glucose) that forces the cells to obtain their energy from oxidative phosphorylation (see below) [6, 20, 52, 53]. By using *Coq7* KO cells, which die in the absence of glucose, we were able to carry out a high-throughput screen for drugs promoting survival under energy stress conditions. The screen yielded only inhibitors of mTOR signaling. However, further analyses using *Pdss2/Mclk1* double knock-out (DKO) cells revealed that these inhibitors require a minimal amount of mitochondrial respiration to produce survival benefits in CoQ-deficient cells and maybe all cells.

## Materials/subjects and methods

### Chemicals and reagents

The compound libraries used are listed in Table S1. For individual compound testing, OSI-027, AZ20, Torin1, AZD6738, and cycloheximide were purchased from Selleckchem. Rapamycin, carbonyl cyanide 4-(trifluoromethoxy)phenylhydrazone (FCCP), oligomycin, rotenone, antimycin A, coenzyme Q_10_, resazurin, crystal violet, hydroxychloroquine, galactose, 2-deoxy-D-glucose (2-DG), and lipoprotein deficient serum (LPDS) were obtained from Sigma-Aldrich. Cell culture reagents were supplied by Wisent, Inc. unless otherwise specified.

### Cell culture

Mouse embryonic fibroblasts (MEFs) were routinely cultured in high glucose DMEM (Dulbecco’s modified Eagle’s medium) supplemented with 10% fetal bovine serum (FBS; Thermo Fisher Scientific) and 1% antibiotic/antimycotic mix. *Coq7* KO and DKO MEFs were prepared as previously described [11, 54]. All cell cultures were confirmed free of mycoplasma using Mycoplasma PCR Detection Kit purchased from ZmTech Scientific Inc. Galactose medium was prepared with glucose-free DMEM (#11966025; Thermo Fisher) and by adding galactose at the final concentration of 10 mM, 1 mM sodium pyruvate, 10% dialyzed FBS (#26400044; Thermo Fisher), and 1 % antibiotic/antimycotic. Both routine culture medium and galactose medium contain 4 mM L-glutamine. Glucose- and galactose-free medium was composed of DMEM without glucose, pyruvate, and glutamine (#A1443001; Thermo Fisher Scientific), 10% dialyzed FBS, and 1% antibiotic/antimycotic. For viability measurements in galactose or glucose- and galactose-free medium, cells were seeded in high-glucose medium overnight and the medium was changed the following day. Cell viability was measured after 2–4 days. Phase-contrast microscopic images were taken using Olympus CKX41 macroscope with Micropublisher 3.3 RTV digital camera (Qimaging).

### Drug screen

*Coq7* KO cells were seeded in 96-well plates in glucose-containing growth medium to obtain ≈ 90% confluence the following day. The media was replaced with galactose medium the next day after washing with PBS one time, and library compounds were added at concentrations of 5 and 10 µM. Each plate contained quadruplicate wells treated with negative DMSO control (0.1% final concentration) and quadruplicate wells treated with CoQ_10_ (10 µM, final concentration) as a positive control. After 4 days of treatment, cell viability was assayed by the resazurin assay. Hits were identified using a threshold of a >20% increase in viability relative to negative DMSO control. Hit confirmation was performed under the same assay conditions in a dose range of 5–20 µM.

### Cell viability assays

Cells were plated in multi well tissue culture plates, and after incubation overnight, the medium was removed and replaced with 200 µl of fresh test medium with the reagent of interest. For the resazurin assay, at the end of treatment, the medium was removed and fresh growth medium containing resazurin at a concentration of 0.15 mg ml^−1^ was added. After 2 h of incubation at 37 °C/5% CO_2_, the absorbances at 570 and 600 nm were measured by a plate reader (TECAN Infinite M1000). A_600_ (absorbance at 600 nm) was subtracted from A_570_ and experimental samples were compared with control conditions to calculate the percentage reduction relative to untreated or vehicle-treated controls. For the crystal violet assay, at the end of treatment, the medium was removed, and cells were washed with PBS once before adding 150 μl of crystal violet solution [0.05% (w/v) crystal violet, 1% formaldehyde, and 1% methanol in 1X PBS] to each well. The plates were incubated at room temperature for 20 min with gentle agitation, after which all traces of dye were removed with distilled water. For quantification, the stain was solubilized with acetic acid (10%) after air-drying, and absorbance was measured in a plate reader (TECAN Infinite M1000) at 590 nm.

### Seahorse extracellular flux assays

Mitochondrial respiration and glycolysis rates of intact cells were determined using a Seahorse Bioscience XFe24 Extracellular Flux Analyzer as described previously [20, 54]. Briefly, cells were seeded at a density of ~30,000 per well, and prior to the measurements, cell medium was replaced with Seahorse XF base medium (supplemented with 10 mM galactose, 2 mM Glutamax, and 1 mM sodium pyruvate) and allowed to equilibrate for 1 h at 37 °C in a non-CO_2_ incubator before the start of the assay. Baseline respiration was measured prior to addition of oligomycin (1 μg/ml). FCCP (0.8 μM) was then injected to assess the maximal respiratory capacity, and non-mitochondrial oxygen-consumption rate (OCR) was measured after addition of rotenone/antimycin A (1 μM/5 μM). Four mitochondrial respiration parameters were determined: basal (baseline respiration minus rotenone/antimycin A post injection respiration), maximal (difference between FCCP stimulated minus rotenone/antimycin A post injection respiration), ATP production-linked (baseline respiration minus oligomycin-insensitive respiration), and spare respiratory capacity (FCCP-stimulated respiration minus baseline respiration). Glycolysis rate was measured using Seahorse XF Glycolysis Stress Test Kit. Data were normalized to protein content using a BCA assay (Thermo Fisher). For OCR measurements under the condition of depletion of lipoproteins, cells were cultured in the medium containing LPDS instead of regular FBS for 5 days before the Seahorse assay.

### Western blot

Cells were lysed in RIPA buffer (Cell Signaling Technology) and protein levels were measured via a BCA assay. About 20 µg of protein was loaded on a 16% SDS-PAGE gel and transferred to a 0.2 μm PVDF membrane (Bio-Rad). Proteins were detected with a rabbit anti-LC3B antibody (1:2000; #2775; Cell Signaling Technology) and an anti-GAPDH antibody (1:4000; #2118; Cell Signaling Technology) as a loading control. Blots were developed with ECL substrates (Froggabio Inc.) followed by exposition to X-ray film.

### Statistical analysis

Appropriate statistical tests were conducted using statistical software GraphPad Prism 8.0 (GraphPad Software, Inc.), with data meeting the assumptions of the tests. Sample sizes were chosen based on similar studies to ensure adequate power to detect a pre specified effect size. All quantitative results are expressed as mean ± standard error of the mean (S.E.M.) or ± the standard deviation (S.D.) as indicated. For viability assays, cell wells were randomly divided into control and experimental groups. No blinding was used because it is not applicable. All data were included in the analyses. Data variations within each group were estimated and similar between statistically compared groups. *p* < 0.05 was used as the cut-off for significant differences of group means.

## Results

### Identification of mTOR inhibitors in a screen for compounds that allow for the survival of CoQ-deficient *Coq7* KO cells under conditions of energy starvation

Cells lacking COQ7 make only the biosynthetic intermediate demethoxyubiquinone (DMQ) [7, 9, 11, 12]. CoQ is required for mitochondrial respiration and these cells therefore cannot survive in a medium where glucose is replaced by galactose [11]. Galactose enters glycolysis by being converted to glucose-1-phosphate, which considerably slows ATP production by glycolysis. Thus, cells to which only galactose is provided must rely on mitochondrial energy metabolism [55].

**Fig. 1:**
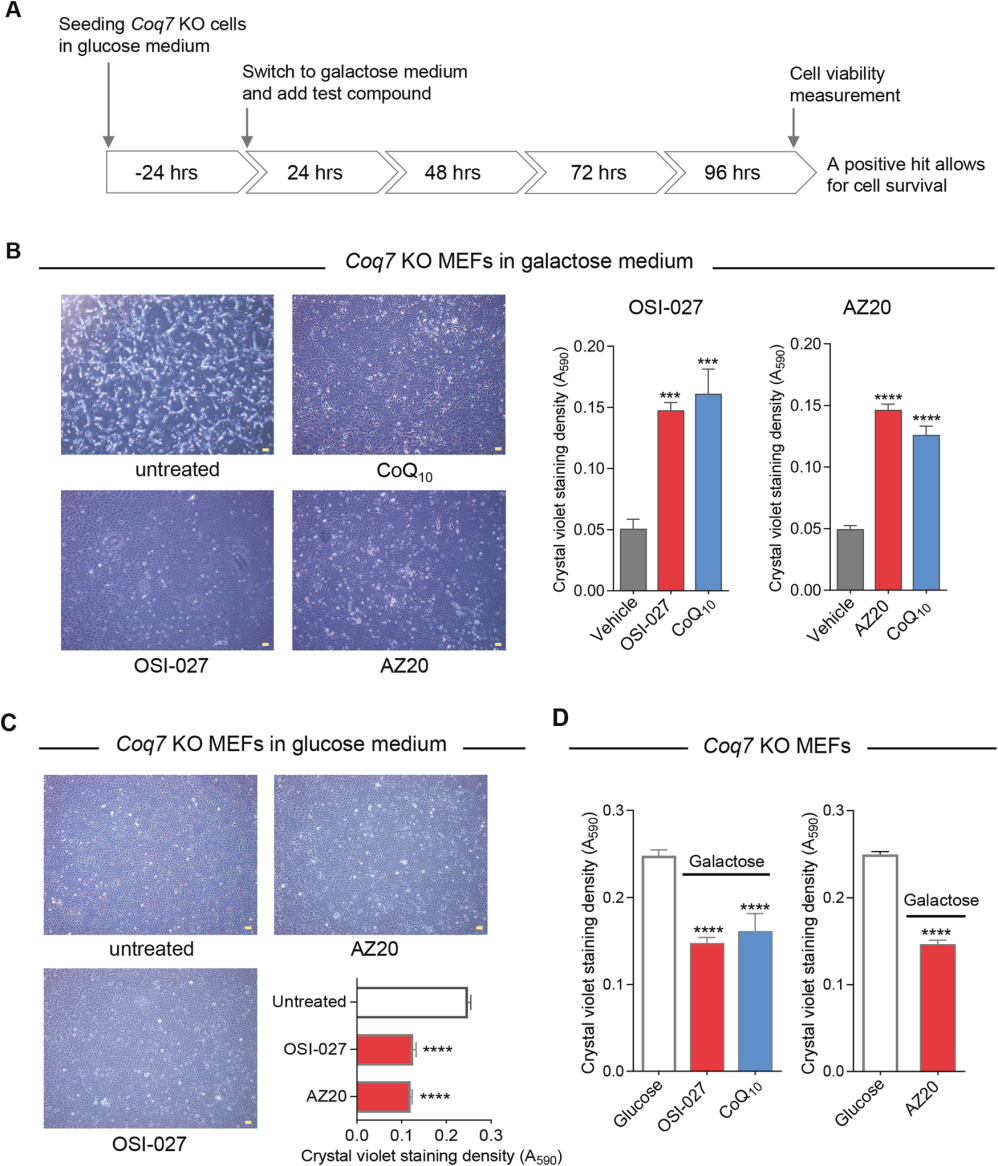
Identification of compounds that enhance the survival of *Coq7* KO cells in galactose medium. **A** Screening strategy. Primary screening of compound library was conducted with *Coq7* KO mouse embryonic fibroblasts (MEFs) and test compounds that increase the survival of *Coq7* KO cells in galactose medium were identified as screen hits. **B** OSI-027 and AZ20, two of the compounds identified in the screen, showed a positive effect on the survival of *Coq7* KO cells in galactose medium. Cell viability was examined after 4-day culture in galactose medium. Phase-contrast microscopy images are shown on the left, and cell growth and survival measured by using the Crystal violet (CV) staining assay is on the right. DMSO is the vehicle control. **C** OSI-027 and AZ20 treatments cause no cell death but decrease CV staining. **D** OSI-027 or AZ20-treated﻿ *Coq7* KO cells in galactose showed a lower CV staining intensity compared with the cells grown in glucose medium. The following doses were used in all experiments unless otherwise stated: OSI-027: 5 µM, AZ20: 10 µM, and CoQ_10_: 10 µM, which was used as a positive treatment control. Scale bars = 100 µm. Data are presented as means ± SEM for *n* = 4–8. ***p < 0.001 and ****p < 0.0001, assessed by one-way ANOVA followed by Dunnett’s multiple comparison test or *t*-test versus the untreated or glucose-grown controls.

To identify compounds that can alleviate the consequences of CoQ deficiency, we screened for compounds that promote the survival of *Coq7* KO cells in media with galactose only (Fig. 1A). Using retroviral *Cre* as described previously [11], *Coq7* KO cells were generated by excising *Coq7* in mouse embryonic fibroblasts (MEFs) extracted from *Coq7*^*fl/fl*^ mice. *Coq7* KO cells show no growth defect in standard glucose medium, but die within 4 days after switching to glucose-free medium supplemented with galactose and dialyzed FBS (hereafter called galactose medium). Dialyzed FBS ensures complete depletion of glucose.

Compounds were screened at 5 and 10 μM and those that led to a >20% increase in viability were considered candidate hits. For screening, we used the resazurin method to score viability for its simplicity and high sensitivity. Potential mechanisms for the rescue of lethality include: (1) boosting of respiration via CoQ-related, or -unrelated mechanisms. This could in principle be achieved by boosting DMQ production (see below) or by altering the physicochemical properties of mitochondrial membranes to allow for more efficient electron transport. (2) Sparing of energy utilization. (3) Blocking of active cell death mechanisms, whether apoptotic or necrotic.

In total, we screened approximatively 8000 compounds from four libraries (Table S1): (1) 1018 FDA-approved and cell-permeable compounds, (2) 1902 bioactive compounds, including inhibitors, (3) 94 compounds with redox properties, and (4) a diversity compound library with no indexed activities. The screen, which included two re-tests, yielded 17 hits, which can be separated into two distinct groups: compounds acting on the mammalian target of rapamycin (mTOR) signaling pathway, and corticosteroids (Table S2). The first group includes 5 selective mTOR inhibitors, 3 phosphoinositide 3-kinase (PI3K) inhibitors, and AZ20, which is the first reported inhibitor of ATR (kinase ataxia telangiectasia and Rad3-related protein) (Fig. S1A). PI3K acts upstream of the mTOR pathway and its inhibition inhibits mTOR signaling. As to AZ20, it is also known as inhibiting mTOR, although it has been developed as an ATR inhibitor [56]. Among the glucocorticoids identified, fluocinonide was the most potent at the screening dosages (5 and 10 µM). It was further tested at concentrations of 2.5–20 µM. However, despite measurable rescue, no concentration of glucocorticoid could entirely prevent cell death based on microscopic observation of cells following treatment (Fig. S1B). Glucocorticoids have been shown to repress mTOR signaling [57, 58]. We currently hypothesize that their partial activity is also mediated by the mTOR pathway. However, given the weak effects observed, we did not attempt to investigate this further in our system.

We focused our study on OSI-027, one of the selective mTOR inhibitors, and AZ20, because it is expected to be mainly an ATR inhibitor [56]. OSI-027 is a dual inhibitor of mTORC1 and of mTORC2 with high selectivity for mTOR relative to PI3Kα, PI3Kβ, PI3Kγ, and DNA-PK [59]. As shown in Fig. 1B, in the presence of OSI-027 or AZ20, *Coq7* KO MEFs remained viable after 4 days of culture in galactose medium, in contrast to vehicle-treated control. The rescue efficiency was almost as good as with supplementation of exogenous CoQ_10_.

Of note, the graphs in Fig. 1B show cell viability measurements determined by crystal violet (CV) staining as described in “Materials and Methods”. The resazurin assay for screening could in principle be affected by changes in cell metabolism. Therefore, we also used CV to score viability and biomass in subsequent studies of the compounds. CV binds to proteins and DNA in cells, and as such, can be used to quantify a viable adherent cell population.

While no cell death/detachment was observed, the CV assay results indicated a significant lower biomass of *Coq7* KO cells after treatment with OSI-027 or AZ20 in standard glucose medium (Fig. 1C). This is expected since inhibition of mTOR is known to slow down cell growth and metabolism and decrease protein synthesis [60]. In fact the same effect was observed in wild-type cells, as well as slower growth in galactose than in glucose medium (Fig. S2). Thus, the galactose-grown *Coq7* KO cells rescued by OSI-027 or AZ20 showed a reduction of cell mass compared with their glucose-grown untreated control, although by visual observation they appeared fully rescued (Fig. 1B, D). Of note, CoQ_10_-treated *Coq7* KO cells in galactose also showed lower cell mass yield compared with glucose-grown controls (Fig. 1D).

### Rescue of cell death induced by complete sugar deprivation

We sought to determine whether the mTOR inhibitors identified in our screen protected against other energetic stresses besides low mitochondrial respiration from CoQ deficiency. We tested the effects on complete sugar deprivation, which induces cell death through multiple mechanisms involving loss of ATP, ER stress, metabolic oxidative stress, and apoptosis activation [61]. We used glucose-free DMEM supplemented with dialyzed FBS (hereafter called glucose- and galactose-free medium). The glucose-free DMEM contains no glutamine or pyruvate. Glutamine enters the mitochondria through the glutaminolysis pathway where it is converted to glutamate and further metabolized to α-ketoglutarate, which enters the Krebs cycle to be oxidized, yielding ATP and NADH [62]. Therefore, glutamine can serve as an energy metabolite for respiration as well as carbon source [63]. Exogenous pyruvate also serves as a direct TCA cycle substrate, besides replenishing NAD+. Increased dependence on glutamine for cell survival after glucose withdrawal has been demonstrated previously, mainly in cancer cells [64–66]. In accordance, we found that wild-type MEFs as well as *Coq7* KO cells die within 2 days in the glucose- and galactose-free medium, but supplementation with glutamine or pyruvate prolongs cell survival, with a larger rescuing effect seen with glutamine (Fig. S3).

**Fig. 2:**
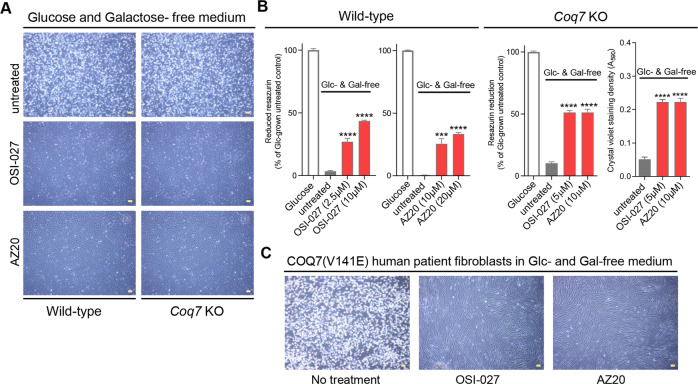
Rescue of cell death in the absence of glucose and galactose. **A**, **B** Phase-contrast microscopy images and cell viability results. The results shown are after 2 days of culture in the medium lacking sugar sources (glucose and galactose). Glc: glucose, Gal: galactose. Means ± SEM are shown (*n* = 4). ***p < 0.001, ****p < 0.0001, assessed by one-way ANOVA followed by Dunnett’s multiple comparison test versus untreated control in the same growth condition. **C** Microscopy images of patient skin fibroblasts carrying the COQ7(V141E) mutation. Pictures were taken after 2-day culture in glucose- and galactose-free medium. OSI-027, 5 µM. AZ20, 20 µM. Scale bars = 100 µm.

For both wild-type and *Coq7* KO cells, we found that ISO-027 and AZ20 prevented cell detachment or loss after 2 days of glucose- and galactose-free culture with dialyzed FBS (i.e., without glutamine or pyruvate), while all untreated cells had died (Fig. 2A, B and Fig. S4). These compounds also could rescue sugar-depletion death in human skin fibroblasts that harbor a homozygous V141E mutation in *COQ7* (Fig. 2C). These cells have a partial but severe deficiency of CoQ_10_ [20,52]. Thus, these compounds can prevent death from the absence of external energy source, such as glucose, galactose, and glutamine, in mouse and human *Coq7* cells that have severely impaired mitochondrial function.

### The effects of rapamycin and Torin 1 on the survival of *Coq7* KO cells in galactose medium

**Fig. 3:**
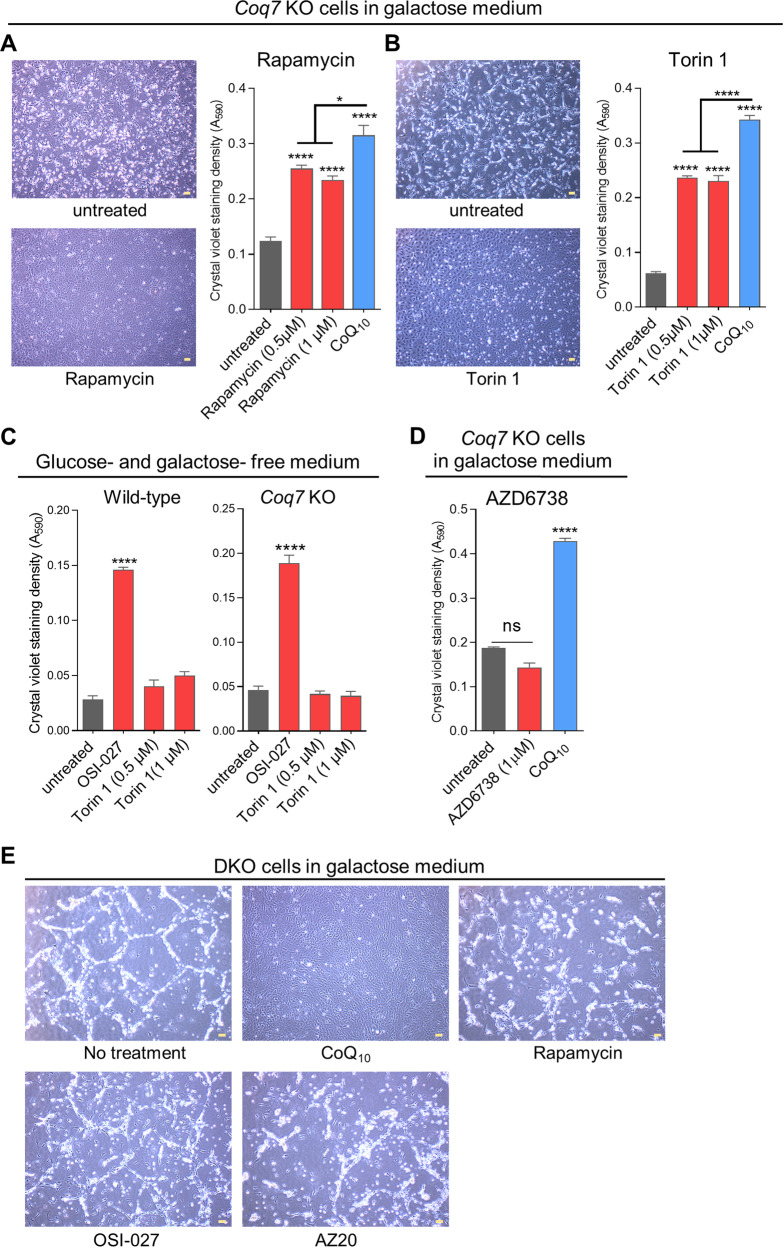
Effects of rapamycin, Torin 1, and AZD6738 on cell death induced by glucose withdrawn. **A**, **B** Viability measurement of *Coq7* KO cells by CV staining after 4-day culture in galactose medium in the presence of the tested compounds. CoQ_10_ (10 µM) was included as a positive control. **C** Torin1 was ineffective to improve cell survival in glucose- and galactose-free medium. OSI-27 (5 µM) was included as a positive control. **D** Viability measurement of *Coq7* KO cells by CV staining after 4-day culture in galactose medium in the presence of AZD6738. Error bar is SEM of 4 biological replicates. *p < 0.05, ****p < 0.0001, assessed by one-way ANOVA followed by Tukey’s multiple-comparison test. ns: not significant. **E** OSI-027 and AZ20 failed to rescue the death of *Pdss2-Coq7* double-KO (DKO) cells in galactose medium. Shown are representative images taken 2 days after switching to galactose medium. CoQ_10_ treatment, resulting in almost full rescue, was used as a positive control. The presence of OSI-027 (10 µM), AZ20 (20 µM), and rapamycin (1 µM) did not prevent the death of DKO cells in galactose medium. Scale bars = 100 µm.

Rapamycin, which is best known for inhibiting mTORC1, was identified in the screen. We retested rapamycin over a wider range of doses, from 0.25 µm to 10 µM, and found rescue at dosages ≥ 0.5 µM (Fig. 3A). Torin 1, a potent inhibitor for both mTORC1 and mTORC2 [67, 68], was not present in the libraries. We found it to be effective in protecting *Coq7* KO cells. However, rapamycin and Torin 1 appeared to be somewhat less effective than OSI-027 and AZ20 (Fig. 3A, B) (compare with Fig. 1B). However, surprisingly, Torin 1 had no effect on cell death from full glucose and galactose deprivation on either wild-type or *Coq7* KO MEFs (Fig. 3C and Fig. S5).

### The ATR inhibitor AZ20 rescues *Coq7* KO cell growth by inhibiting mTOR

We sought to determine whether the rescue by AZ20 was the result of inhibition of mTOR and/or of ATR. For this, we tested AZD6738, a potent and selective ATR inhibitor [69], but observed no effect on the death of *Coq7* KO cells in galactose or glucose- and galactose-free medium (Figs. 3D and S6). These findings suggest that it is the inhibition of mTOR that is crucial for the effect of AZ20 on cell survival under conditions of energy starvation.

### mTOR inhibition cannot increase the survival of *Pdss2-Coq7* double KO cells during glucose deprivation

Above, we have shown that *Coq7* KO cells can be rescued by mTOR inhibition even in the absence of fermentable carbon sources and other nutrients that can be utilized to generate energy (e.g., glutamine and pyruvate), and this is despite their lack of CoQ and impaired mitochondrial respiration. We decided to explore if this relates to the presence of the intermediate DMQ, whose functional significance for the mutant phenotype is not fully elucidated [7, 9, 11, 12]. We generated MEFs that lack the expression of both the *Coq7* and *Pdss2* genes [54]. Both genes are necessary to make CoQ, but loss of *Pdss2* also prevents the synthesis of DMQ [54]. We found that, as expected, *Pdss2* and *Coq7* double-KO cells (DKO) cannot survive in galactose medium but can be rescued by supplementation with CoQ_10_ (Fig. 3E) [54]. We tested OSI-027, AZ20, and rapamycin for their effect on DKO cells in galactose, but all were equally ineffective (Fig. 3E).

### *Coq7* KO cells display a higher respiration rate than *Pdss2/Coq7* DKO cells, thanks to electron transport by demethoxyubiquinone (DMQ)

**Fig. 4:**
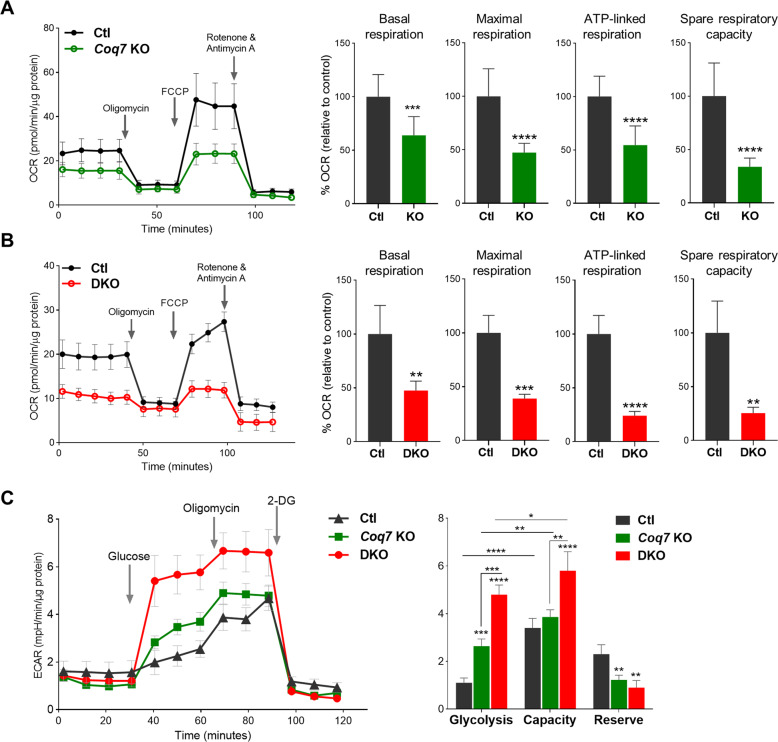
A comparison of mitochondrial respiration and glycolytic activity between *Coq7* KO and DKO cells. **A**, **B** Representative oxygen-consumption rate (OCR) traces are shown on the left and bar graphs show various respiratory parameters calculated using the raw traces. Values were normalized to total protein and expressed as percentage of their respective controls (Ctl) in the bar graphs. Significance was analyzed by Student’s *t*-test. **C** Glycolytic rate measurement. Sequential injection of glucose and oligomycin and 2-deoxyglucose (2-DG) was used to calculate glycolytic capacity and reserve. Significance was calculated by one-way ANOVA followed by Tukey’s post hoc test. *p < 0.05, **p < 0.01, ***p < 0.001, and ****p < 0.0001. Error bar is SD (*n* = 4–10). Ctl in C was *Coq7* floxed but not recombined cells.

We directly measured respiration rates to explore why *Coq7* KO cells, but not DKO cells, can be rescued by mTOR inhibition in the absence of glucose. The single- or double-floxed cells infected with empty retrovirus were used as controls. We found that mitochondrial oxidative phosphorylation, including basal respiration, maximal respiration, spare respiratory capacity, and ATP production, was severely compromised in both CoQ-deficient mutants, but to a greater extent in DKO cells (47.5% vs. 64.0%, 39.0% vs. 47.5%, 24.1% vs. 54.5%, and 25.4% vs. 33.8%, respectively) (Fig. 4A, B). In line with this, DKO cells were found to have a greater increase in glycolytic rate and a lower glycolytic reserve relative to *Coq7* KO cells, indicating that they operated at maximal glycolytic rate as a compensation for the total loss of mitochondrial ATP production (Fig. 4C).

**Fig. 5:**
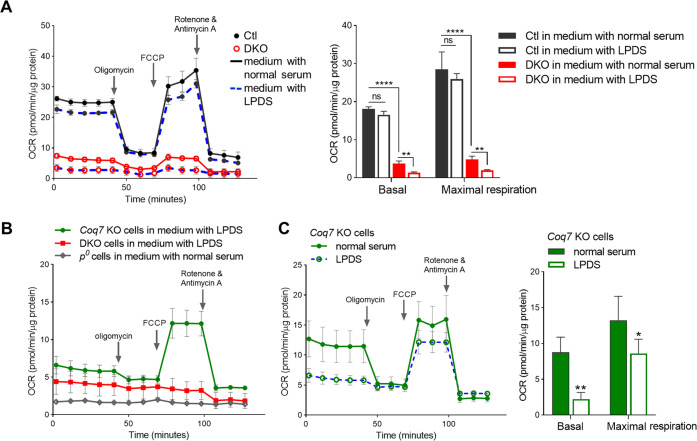
OCR measurements after 6 days of culture in the presence of LPDS. **A** Replacement of regular FBS with LPDS resulted in loss of respiration in DKO cells, while it has a minimal effect on wild-type control cells. **B** OCR comparison of *Coq7* KO, DKO, and rho-zero (*ρ*^0^) cells after culture in medium with LPDS. **C** OCR of *Coq7* KO cells was decreased in LPDS-supplemented medium but with a considerable activity remaining. OCR was expressed in picomoles of O_2_ per minute per microgram of protein. Data show mean ± SD (*n* = 3–7) and assessed by Students’ *t*-test or two-way ANOVA followed by post-hoc Tukey’s test where appropriate. ^*^p < 0.05, ^**^p < 0.01, and ^****^p < 0.0001.

We wondered whether the residual respiration in DKO cells resulted from CoQ uptake from the medium. We therefore measured mitochondrial respiration after replacing the regular FBS with a lipoprotein-deficient serum (LPDS) (S5394, Sigma). Depletion of lipoproteins from serum removes CoQ, as we verified (Fig. S7). After 6 days of culture in medium with LPDS, DKO cells showed a complete loss of response to the ETC modulators oligomycin, FCCP, and rotenone/antimycin A (Figs. 5A and S8), indicating the loss of a functional ETC. In fact, the OCR trace of DKO cells cultured in LPDS medium became very similar to that of mtDNA-deficient Rho-zero (ρ0) cells (Fig. 5B), which lack aerobic respiration altogether [70]. In contrast, culture in LPDS medium for 6 days did not abolish the respiration of *Coq7* KO cells. The respiration of LPDS-grown *Coq7* KO cells was decreased, but significant activity was retained, especially under uncoupled conditions (Fig. 5C). The higher sensitivity of DKO cells to LPDS is consistent with a total reliance on exogenous CoQ for mitochondrial electron transport. We conclude that the DMQ that is present in *Coq7* KO cells can partially functionally replace CoQ as a mitochondrial electron carrier in the respiratory chain.

### Requirement of respiration for pyrimidine synthesis

Last, it is worth noting that cells without a functional ETC, such as ρ0 cells, can grow glycolytically but require uridine supplementation. This is because dihydroorotate dehydrogenase (DHODH), a crucial enzyme in de novo pyrimidine synthesis, depends on a functional respiratory chain and requires CoQ as an electron acceptor [41]. Both *Coq7* KO and DKO cells can grow in routine culture conditions and even, for a few passages, in medium with LPDS, without the need for exogenous addition of uridine to the growth medium. We speculate that this may reflect a minimum requirement for mitochondrial respiratory function to maintain adequate pyrimidine synthesis when the respiratory chain is intact, even when functioning at an extremely low rate because of lack of CoQ.

### The effect of cycloheximide on cell death induced by energy failure

**Fig. 6:**
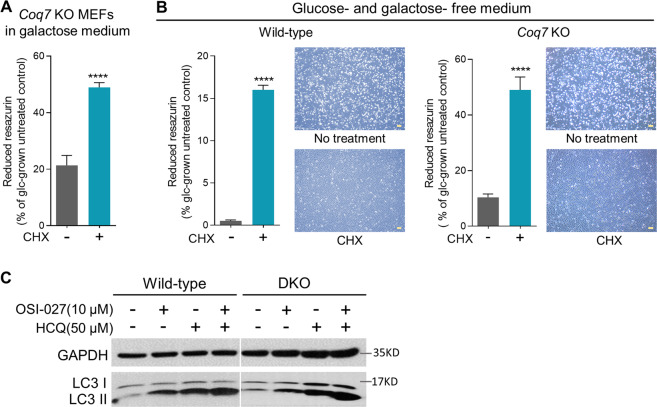
Protective effect of cycloheximide (CHX) on cell death induced by glucose withdrawn. **A** Cell viability measurement of *Coq7* KO cells after 4 days of culture in galactose medium. **B** Viability measurement of wild-type or *Coq7* KO cells after 2 days of culture in the sugar-free medium. Resazurin assay was used for determining cell viability with the results presented as relative to glucose-grown control cells. Error bars represent SEM (*n* = 4). CHX: 3 µM. ^****^p < 0.0001 (one-way ANOVA followed by Tukey’s multiple-comparison test). **C** Western blot of LC3 protein in wild-type and DKO MEFs after 2 days of indicated treatments. HCQ: hydroxychloroquine. Scale bars = 100 µm.

One critical consequence of mTORC1 inhibition is a global decrease of protein synthesis [71]. To determine whether the inhibition of protein synthesis is sufficient to protect cell death from energy failure, we tested cycloheximide (CHX), a well-known protein synthesis inhibitor. Indeed, we found that CHX treatment resulted in an extended survival of *Coq7* KO cells in galactose medium, and also prolonged cell survival in the starvation medium lacking all sugars (Figs. 6A, B). Protein synthesis is the most energy-consuming metabolic process. Rescue by CHX suggests that shutting down energy expenditure is the main mechanism for mTOR inhibition to extend cell survival under energy stress.

### The role of autophagy in rescue by mTOR inhibition

The induction of autophagy is another well-established consequence of mTOR inhibition. Autophagy allows for recycling of essential nutrients and providing substrates for biosynthesis under nutrient-starved conditions [72, 73]. Consistent with previous studies, we detected increased levels of the autophagic marker LC3-II upon OSI-027 treatment in wild-type cells (Fig. 6C) [74]. Microtubule-associated protein light chain 3 (LC3) is an essential component of autophagosomes and includes two forms, soluble LC3-I and a lipidated membrane-bound form (LC3-II). LC3I–LC3II conversion is indicative of autophagosome formation [75]. Accumulation of LC3-II after OSI-027 treatment was not suppressed in the presence of hydroxychloroquine (HCQ), which impairs autophagosome fusion with lysosomes, indicating that the observed increase of LC3-II after OSI-027 treatment reflects increased autophagosome formation rather than decreased autophagosome degradation. In DKO cells, we observed the same effects as in wild-type cells (Fig. 6C), suggesting that autophagy is not a crucial mechanism by which mTOR inhibition prevents cell death from energy crisis in our system.

## Discussion

Our screen allows for the identification of compounds that relieve the deleterious consequences of CoQ deficiency through CoQ-related and -unrelated mechanisms. Strikingly, we identified only PI3K/AKT/mTOR pathway inhibitors. PI3K/AKT/mTOR is a major signaling pathway regulating cell growth, metabolism, and survival during cellular stresses. PI3K and AKT are upstream of mTOR and their inhibition prevents activation of mTOR [71]. mTOR is a central nutrient and energy sensor in the cell. mTOR functions in the two complexes mTORC1 and mTORC2. Inhibition of mTORC1 leads to the inhibition of anabolic processes (such as protein and lipid synthesis) and triggers catabolism to replenish ATP. The best inhibitors also inhibit mTORC2 to avoid an activation of mTORC2 via a negative feedback loop that can counteract the effect of mTORC1 inhibition [44].

We identified several mTORC1/2 dual inhibitors, PI3K inhibitors, and AZ20, an inhibitor of ATR kinase that also inhibits mTOR. Further analyses showed that OSI-027 and AZ20 not only prolong the survival of *Coq7* KO cells in galactose medium but also protect against the energetic stress in sugar-free medium. We thus show that mTOR inhibition is beneficial for cell survival under severe energetic stress due to CoQ deficiency. mTOR inhibition was also effective in a model of pharmacologic inhibition of mitochondrial function [50]. Our findings suggest however that the key downstream effectors promoting cell survival remain to be identified. Indeed, it is unclear why AZ20, which is mostly specific for ATR, is as efficient as OSI-027, and why Torin 1, which is a potent dual inhibitor [67], shows only weak survival-promoting activity on *Coq7* KO cells in galactose but not under restriction of both glucose and galactose.

Inhibition of mTOR with rapamycin has been shown to exert beneficial effects on several RC-deficiency models, including models of Leigh syndrome, mtDNA depletion, and renal disease in *Pdss2*^*kd/kd*^ mice [45, 48, 50]. However, a more recent study found no effect on *Coq9*^*R239X*^ mouse mutants [51]. The discrepancy of the effect in the two CoQ deficiency models is not understood. Compared with rapamycin, OSI-027 has been shown to provide greater antineoplastic effect in solid tumor models in vitro and in vivo [59]. Our study also suggests a higher potency of OSI-027 over rapamycin. Future studies should therefore compare OSI-027 with rapamycin in CoQ-deficiency models.

Intriguingly, we found that the mTOR inhibition is not effective in prolonging the survival of DKO cells against energetic stress. It suggests that lowering energy expenditure from protein synthesis and stimulating autophagy are insufficient to provide either enough energy in the severe absence of mitochondrial respiration, or mitochondrial respiration is needed for other processes beyond energy production that cannot be alleviated by mTOR inhibitors.

Our analyses to understand the differences between *Coq7* KO and DKO cells led us to a definitive demonstration that DMQ can carry electrons in the mammalian respiratory chain. Although DMQ does not seem to support respiration in *S. cerevisiae* [76], in *C. elegans*, the presence of DMQ contributes to the viability of the *clk-1* mutants (*Coq7* ortholog), as mutants completely devoid of endogenous CoQ or DMQ are not viable, even if fed CoQ-replete bacteria [9, 13]. In vitro findings further suggest that DMQ competes with CoQ in the respiratory chain of worms [12]. However, this competition is likely not seriously deleterious as the loss or oxygen consumption in *clk-1* mutants is mild and the presence of DMQ does not contribute to most of the Clk-1 phenotypes [77, 78]. Indeed, missense tRNA suppressors of *clk-1(e2519)* point mutation produced a full rescue of the Clk-1 phenotypes, despite only very little CoQ and an unchanged accumulation of DMQ [77]. In mice, total KO of *Coq7* is embryonically lethal, similar to mutations in other CoQ biosynthetic enzymes [5, 7, 79]. *COQ7* patients have also been reported to have severe clinical manifestations [52, 80]. Thus, DMQ-supported respiration is too low, or deficient in some other way, to permit sufficiently normal cellular function.

## Supplementary information

Supplementary Figure Legends.docx

Figure S1

Figure S2

Figure S3

Figure S4

Figure S5

Figure S6

Figure S7

Figure S8
