# Supplementary material for: Minimal mitochondrial respiration is required to prevent cell death by inhibition of mTOR signaling in CoQ-deficient cells
Source: Cell Death Discov. 2021 Aug 4;7:201. doi: 10.1038/s41420-021-00591-0 (PMC8338951; doi:10.1038/s41420-021-00591-0)
Supplement: Supplementary file 1 — Supplementary Figure Legends.docx [file 41420_2021_591_MOESM1_ESM.docx]

**Supplementary Figure Legends**

**Supplementary Figure 1: mTORC1 pathway Inhibitors Identified in our screen. A)** Viability measurements of *Coq7* knockout (KO) MEFs after 4-day culture in galactose medium with various drug treatments. **B)** Partial rescue of the survival defect of *Coq7* KO cells in galactose by fluocinonide. Cell pictures show the lack of cytotoxicity of fluocinonide at the test dose of 10 µM in glucose medium but it was only partially effective at increasing the survival of *Coq7* KO cells in galactose medium at any dosage. CoQ_10_ (10 µM) was used as a positive control. Cell viability was measured by the resazurin reduction assay and expressed as percentage of the amount of viable cells cultured in the complete glucose-containing medium. Mean values ± the standard error of the mean (SEM) are shown. n=4. Scale bars = 100 µm. **p*<0.05, ***p*<0.01, ****p*<0.001, and *****p*<0.0001 (by one-way ANOVA followed by Dunnett's post hoc test compared to untreated galactose-grown cells).

**Supplementary Figure 2: Effects of mTOR inhibitors on wild-type MEFs. A)** Viability measurement of wild-type MEFs after 4 days of the indicated treatments in standard glucose medium. n=4. **B)** Comparison of cell growth in glucose versus galactose medium. The measurement was carried out after 2 days of culture in the indicated conditions. n=8. Cell viability was measured by the resazurin reduction assay and expressed as percentage of the amount of viable cells cultured in the complete glucose-containing medium. Mean values ± SEM are shown. *****p*<0.0001 (by one-way ANOVA followed by Dunnett's multiple comparison test or Student's *t-*test as appropriate.

**Supplementary Figure 3: Addition of glutamine or pyruvate increases cell survival under glucose deprivation.** Cell appearance was observed, and viability was measured under 2 days of culture in glucose (Glc) - and galactose (Gal) medium. Standard concentrations of L-glutamine (2 mM) and pyruvate (1mM) were used. Resazurin assay was used for determining cell viability, and data are shown as percent of that of glucose-grown wells. Mean ± SEM. n=6. Scale bars = 100 µm. **p*<0.05 and *****p*<0.0001 (by one-way ANOVA with Dunnett's post hoc test compared to untreated wells in Glc- and Gal- free medium).

**Supplementary Figure 4: Photographs of wells stained with crystal violet (CV).** Wild-type MEFs were cultured in glucose- and galactose-free medium for 2 days before stained with CV. Blue stain indicates intact monolayer (cells attached to the plastic). In the presence of OSI-027 (5 µM) or AZ20 (20 µM), more viable cells remained after the sugar-free medium treatment.

**Supplementary Figure 5: Torin 1 is ineffective at inhibiting the cell death in glucose- and galactose-free medium.** Cells were seeded in 12-well plates and cultured for 2 days in the indicated culture conditions before imaging. Drug treatments in glucose medium showed no cytotoxicity at the tested doses. Scale bars = 100 µm.

**Supplementary Figure 6: No effect of AZD6738 on the death of *Coq7* KO cells upon glucose withdrawal. A)** Phase-contrast microscopy images of *Coq7* KO MEFs after 4 days of the indicated treatments. **B**) Toxicity test of AZD6738 in glucose medium showing AZD6738 was not toxic at 1 µM but showed minimal toxicity at 5 µM. Values are mean ± SEM (n=4). ***p*<0.01 (by one-way ANOVA with Dunnett's post hoc test). **C**) Images of *Coq7* KO cells after 2 days of culture under the indicated conditions. Glc: glucose. Gal: galactose. Scale bars = 100 µm.

**Supplementary Figure 7: HPLC traces of CoQ extracts from regular FBS or lipoprotein-deficient serum (Sigma S5394).** CoQ_10_ is detectable in regular FBS but not in the serum that is deprived of lipoproteins.

**Supplementary Figure 8: OCR measurement comparison between DKO cells cultured in medium with regular serum or in medium with lipoprotein-deficient serum (LPDS).** OCR were measured by Seahorse XFe24 Analyzer (Agilent) after culture in the indicated conditions for 6 days. The cell image in **B** shows that DKO cells were viable after 6 days of culture in the medium containing LPDS. Scale bars = 100 µm.

**Supplementary Table 1: List of compound libraries screened.**

| **Compound library name** | **Supplier** | **Number of compounds** | **Key features of the library compounds** |
| --- | --- | --- | --- |
| Bioactive chemical  compounds | Selleckchem | 1902 | Bioactive compounds including active pharmaceutical ingredients, natural products, chemotherapeutic agents, etc. |
| FDA-approved Drug Library | Selleckchem | 1018 | FDA approved drugs |
| SCREEN-WELL REDOX  Library | Enzo Life Sciences | 94 | With defined prooxidant or antioxidant activity |
| ChemBridge PremiumSet  Library | ChemBridge Corp | 5000 | Structurally diverse and with desirable structural features for potential drug candidates |

**Supplementary Table 2: Screen hits and their known activities**

| Compound Name | Target | Information |
| --- | --- | --- |
| OSI-027 | mTOR | Dual inhibitor of mTORC1 and mTORC2 with more than 100-fold selectivity for mTOR versus PI3Kα, PI3Kβ, PI3Kγ or DNA-PK |
| WYE-125132 | mTOR | ATP-competitive inhibitor of mTORC1 and mTORC2; highly selective for mTOR versus PI3Ks or PI3K-related kinases hSMG1 and ATR |
| AZD2014 | mTOR | ATP-competitive mTORC1/2 dual inhibitor, with a high level of selectivity versus other members of the PIKK family |
| PP242 | mTOR | Targeting both mTOR complexes with high selectivity for mTOR versus PI3K |
| Rapamycin | mTOR | Specific allosteric inhibitors of mTOR with preference to mTORC1 |
| CH5132799 | mTOR, PI3K | Selective class I PI3K inhibitor, especially against PI3Kα |
| BKM120 | PI3K | Selective and reversible pan-class I PI3K inhibitor |
| TG100713 | PI3K | Pan-PI3K inhibitor |
| AZ20 | ATM/ATR | Selective inhibitor of ATR kinase with high selectivity versus mTOR |
| Fluocinolone Acetonide | corticosteroid | Corticosteroid that binds to the cytosolic glucocorticoid receptor |
| Halcinonide | corticosteroid | Corticosteroid used in topical preparations as an anti-inflammatory agent |
| Betamethasone dipropionate | corticosteroid | Glucocorticoid steroid with anti-inflammatory and immunosuppressive abilities |
| Betamethasone valerate | corticosteroid | Glucocorticoid steroid with anti-inflammatory and immunosuppressive properties |
| Hydrocortisone | corticosteroid | Steroid hormone produced by the adrenal gland |
| Fluticasone propionate | corticosteroid | Synthetic corticosteroid which is derived from fluticasone used to treat asthma and allergic rhinitis |
| Fluocinonide | corticosteroid | Glucocorticoid steroid used topically as anti-inflammatory agent for the treatment of skin disorders such as eczema |
| Dexamethasone acetate | corticosteroid | Synthetic glucocorticoid used as an anti-inflammatory and immunosuppressant |
