# Supplementary figures and images for: Minimal mitochondrial respiration is required to prevent cell death by inhibition of mTOR signaling in CoQ-deficient cells
Source: Cell Death Discov. 2021 Aug 4;7:201. doi: 10.1038/s41420-021-00591-0 (PMC8338951; doi:10.1038/s41420-021-00591-0)

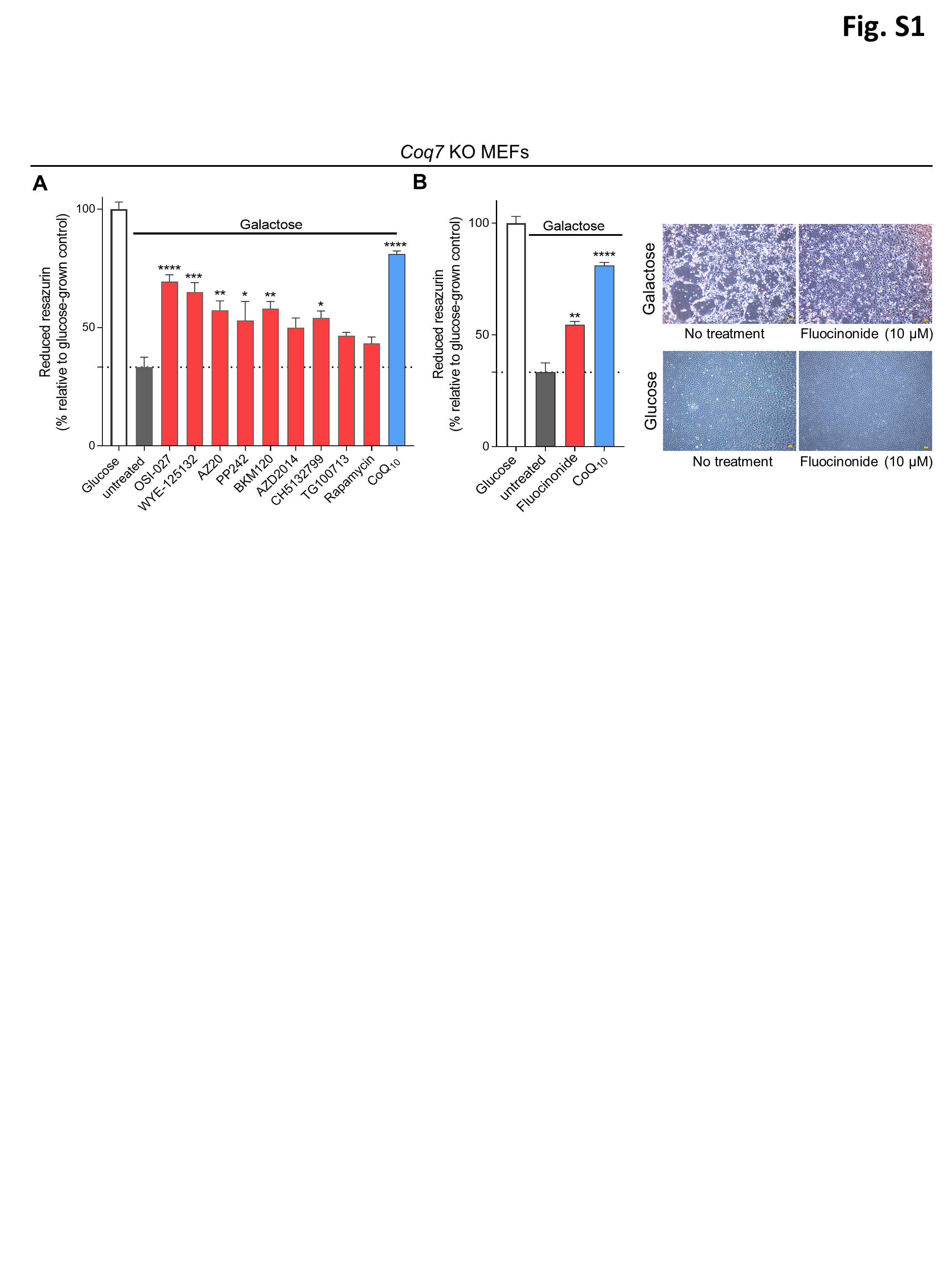

Supplement: Supplementary file 2 — Figure S1 [file 41420_2021_591_MOESM2_ESM.tif]

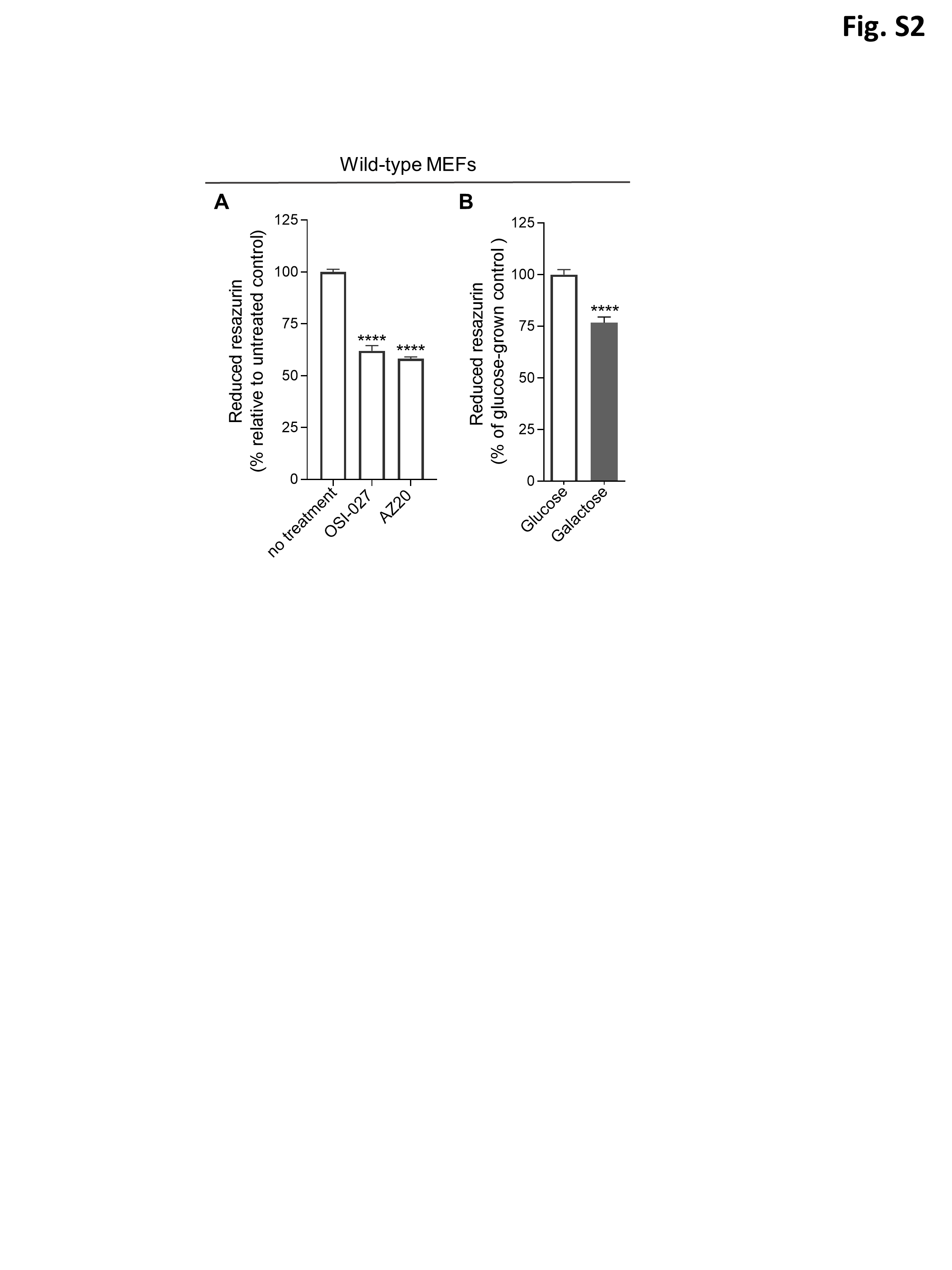

Supplement: Supplementary file 3 — Figure S2 [file 41420_2021_591_MOESM3_ESM.tif]

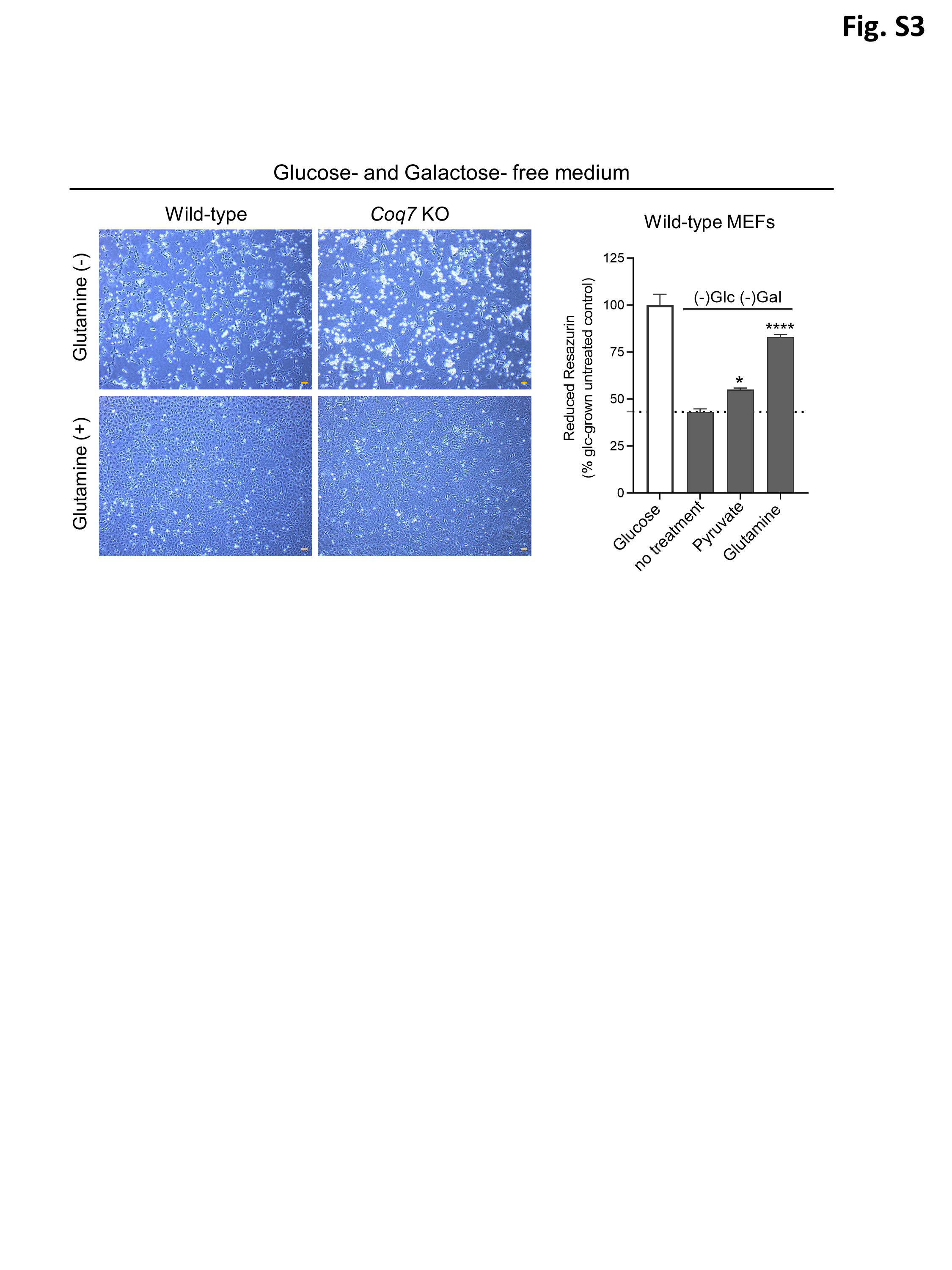

Supplement: Supplementary file 4 — Figure S3 [file 41420_2021_591_MOESM4_ESM.tif]

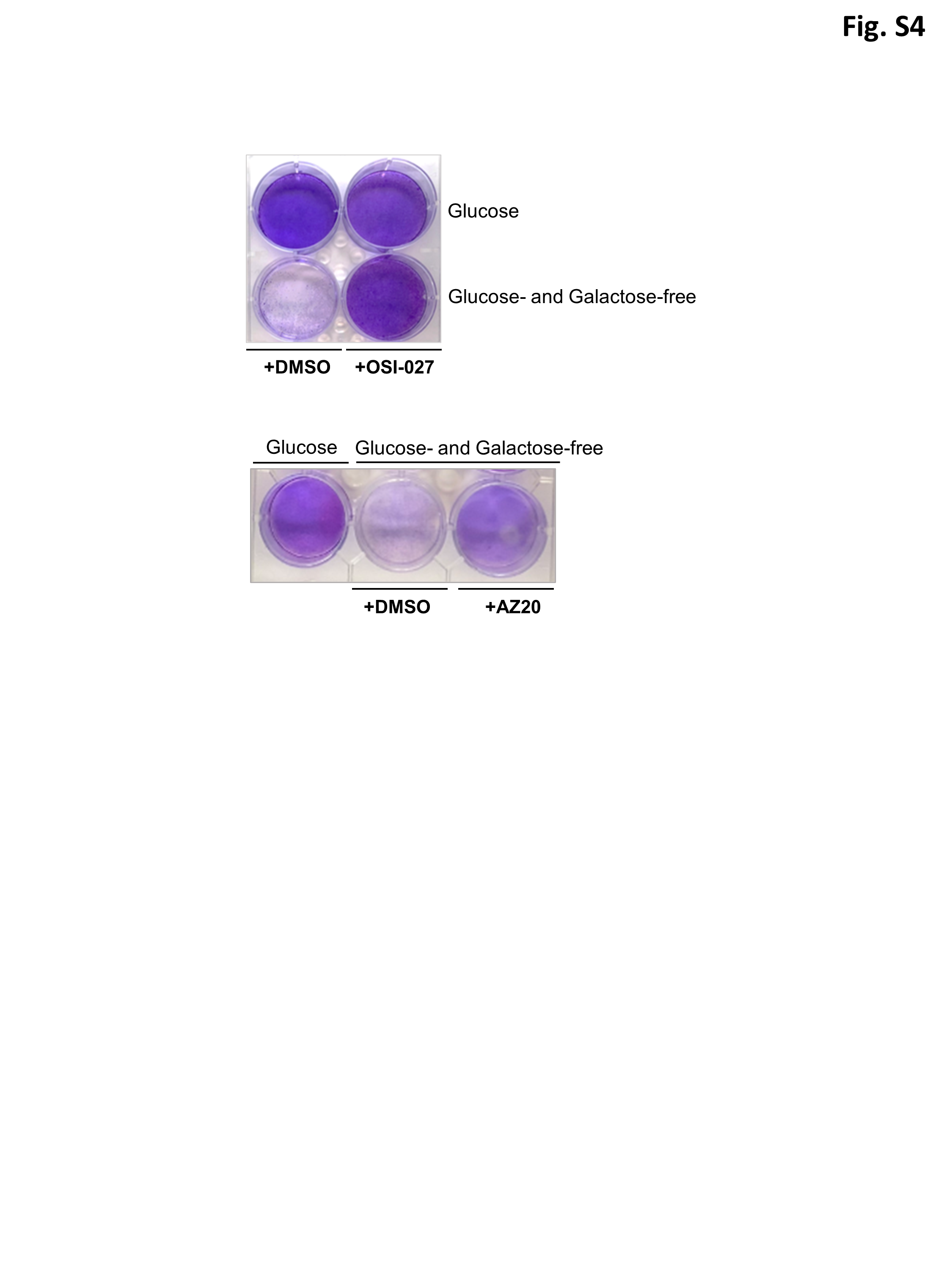

Supplement: Supplementary file 5 — Figure S4 [file 41420_2021_591_MOESM5_ESM.tif]

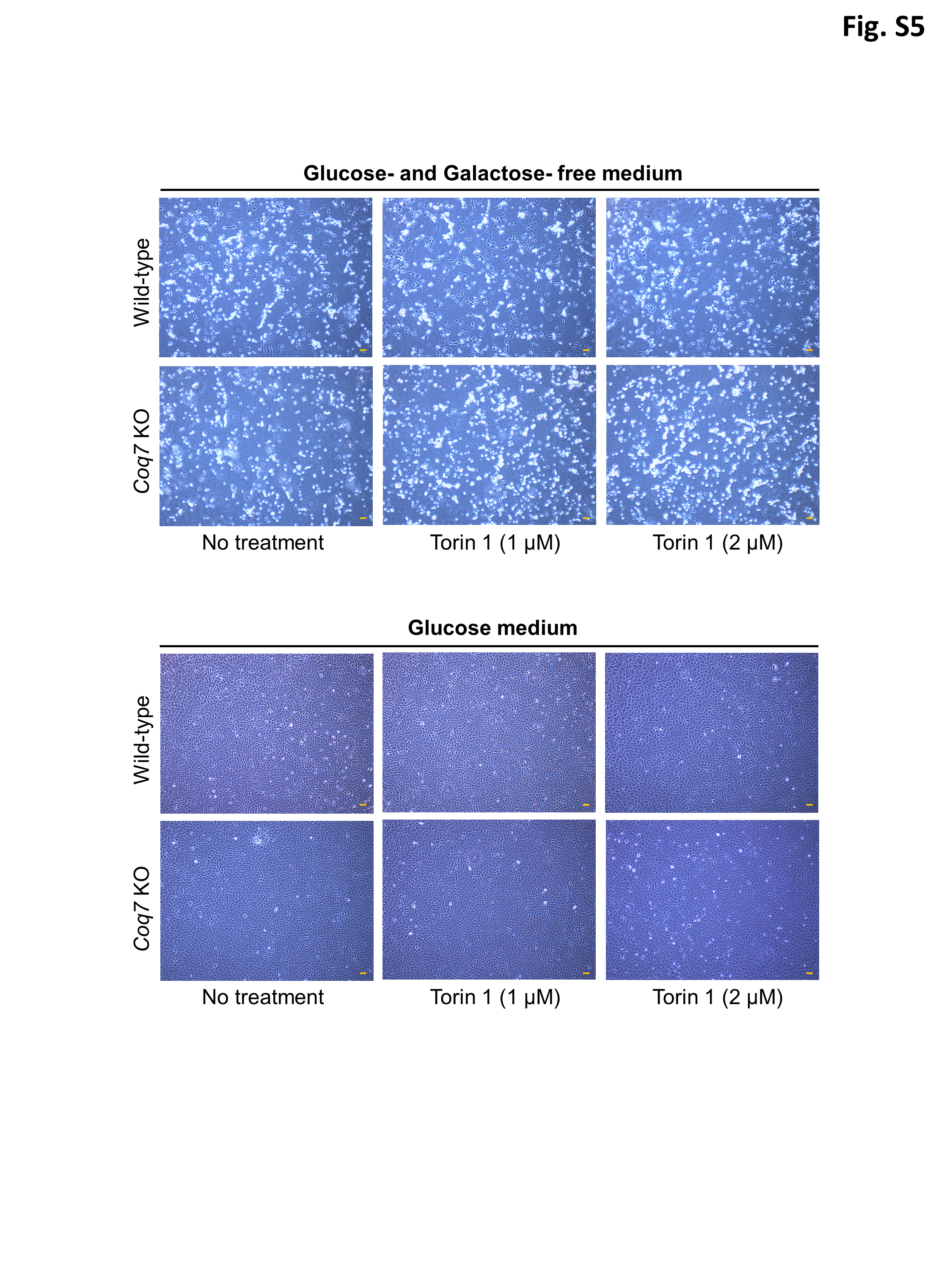

Supplement: Supplementary file 6 — Figure S5 [file 41420_2021_591_MOESM6_ESM.tif]

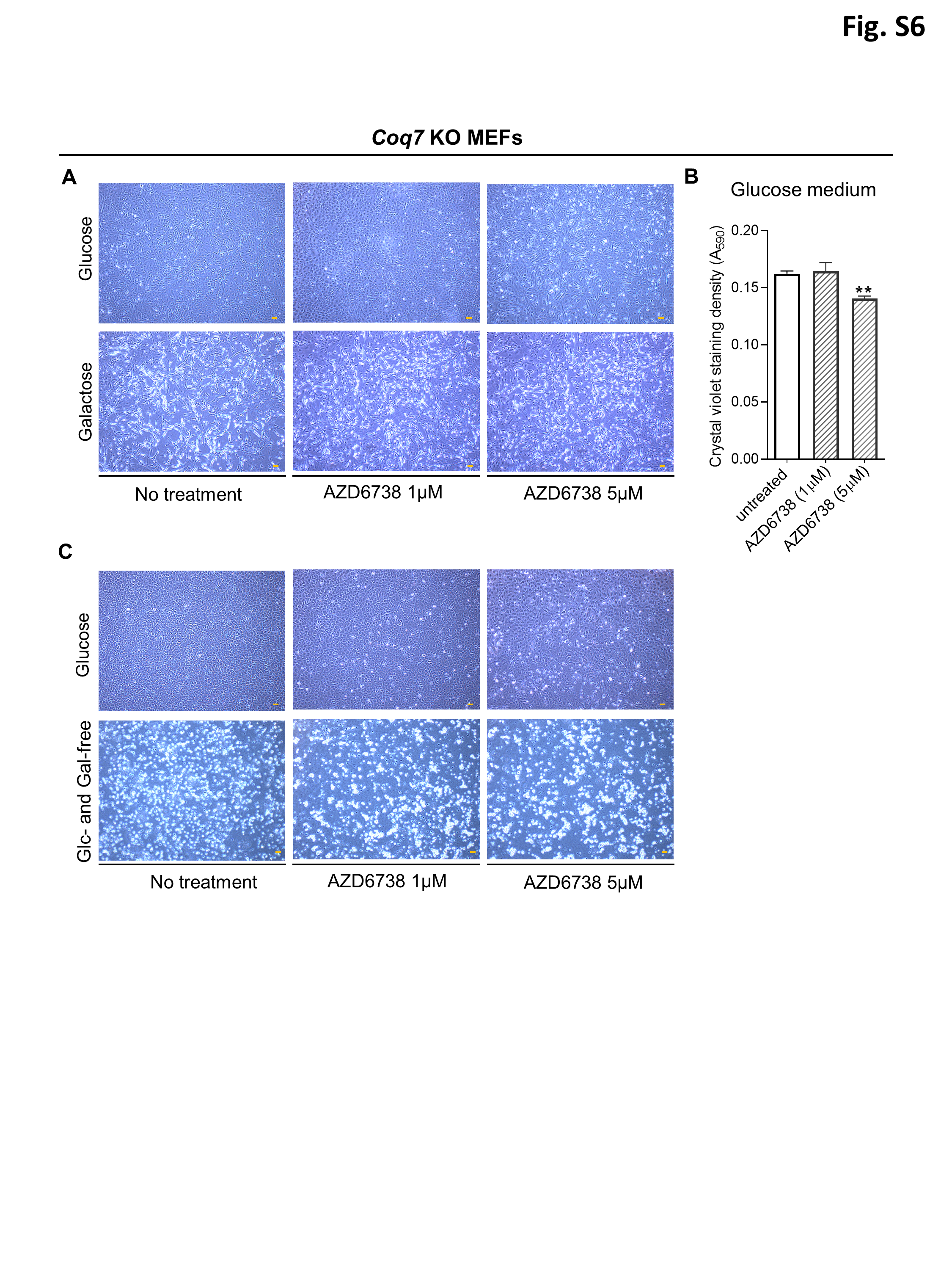

Supplement: Supplementary file 7 — Figure S6 [file 41420_2021_591_MOESM7_ESM.tif]

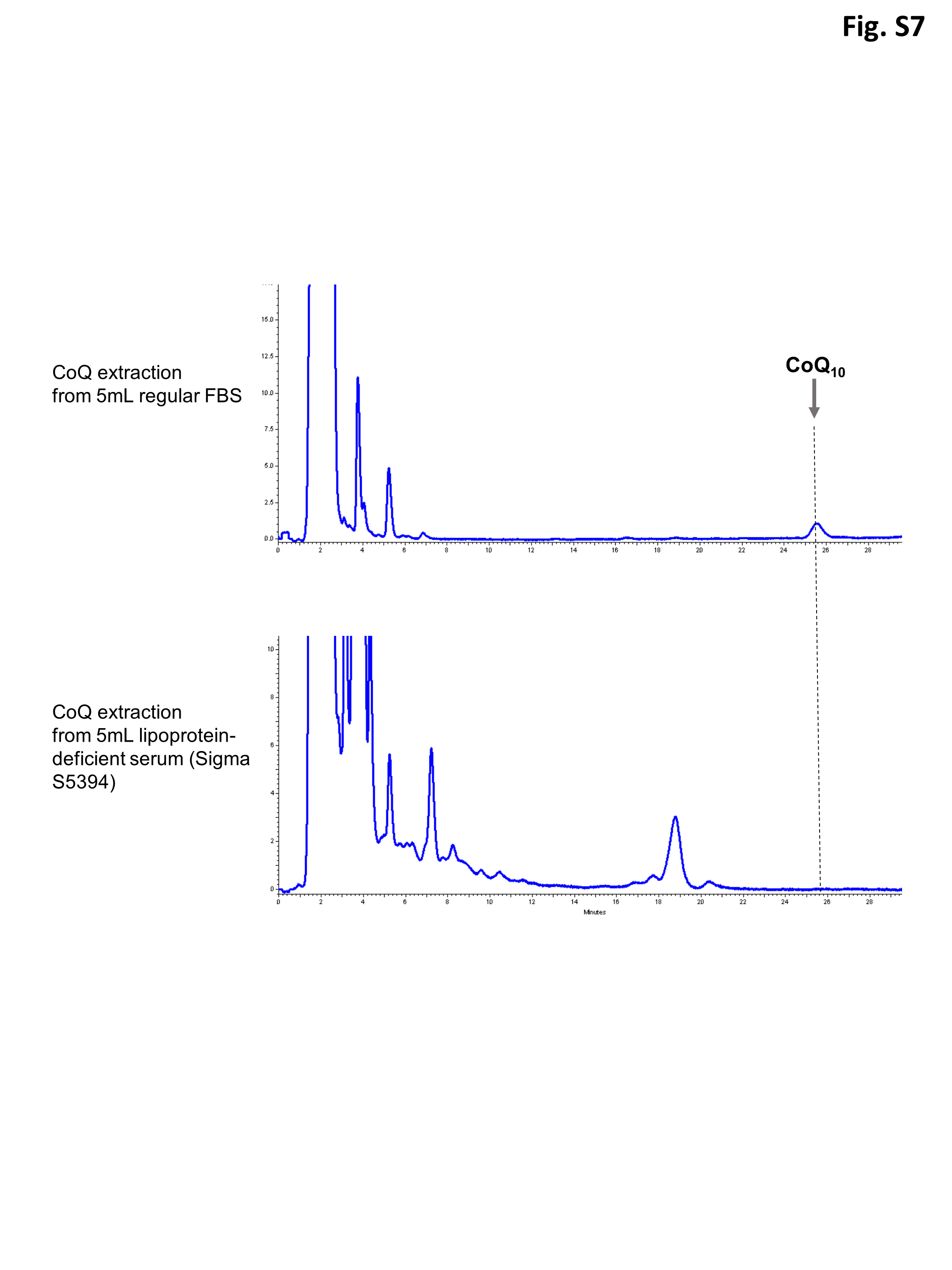

Supplement: Supplementary file 8 — Figure S7 [file 41420_2021_591_MOESM8_ESM.tif]

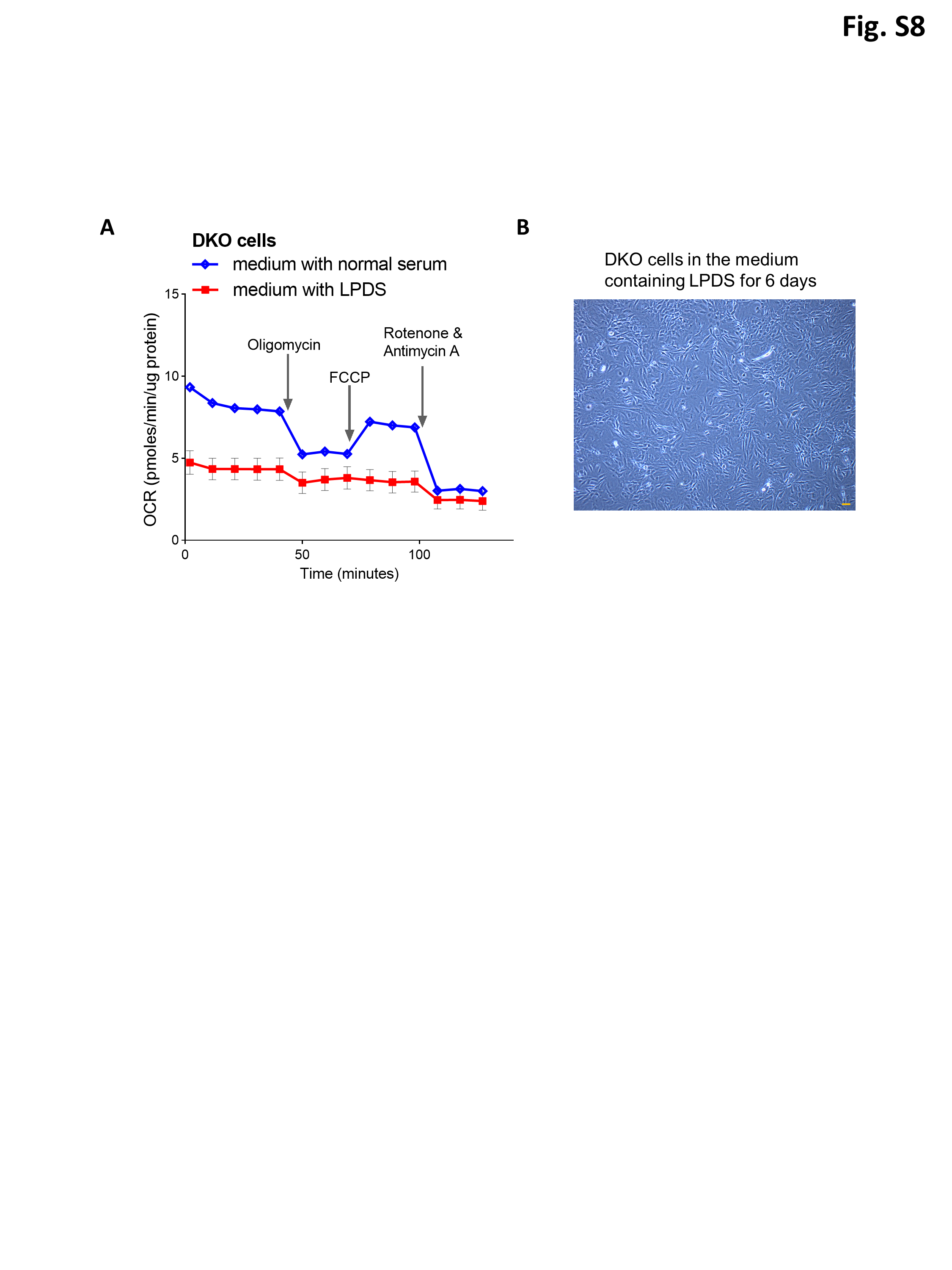

Supplement: Supplementary file 9 — Figure S8 [file 41420_2021_591_MOESM9_ESM.tif]
